# Diagnosis and Treatment of Psychiatric Comorbidity in a Patient with Charles Bonnet Syndrome

**DOI:** 10.1155/2014/195847

**Published:** 2014-11-06

**Authors:** Jasper J. Chen

**Affiliations:** ^1^Behavioral Health Services, Cheyenne Regional Medical Center, Cheyenne, WY 82001, USA; ^2^Department of Psychiatry, Geisel School of Medicine at Dartmouth College and Dartmouth-Hitchcock Epilepsy Center at Dartmouth-Hitchcock Medical Center, Lebanon, NH 03756, USA

## Abstract

*Background*. A significant proportion of patients with neurological disorders may have comorbid psychiatric symptomology, which may be managed by primary outpatient neurologists. Referral to their psychiatric colleagues is mediated by available consultation-liaison units and according to clinical opinion. *Aims of Case Report*. We present the case of a patient whose initial referral to epilepsy clinic led to a workup which ultimately diagnosed her with nonepileptic seizures (NES). In the course of her follow-up, she developed intractable headaches, and worsening mood symptoms and eventually exhibited Psychotic Features for which psychiatry became coinvolved in her care. Major Depression with Psychotic Features and Charles Bonnet syndrome were considered as a likely comorbid diagnoses. Her pharmacologic management on venlafaxine and quetiapine eventually caused substantial amelioration of her psychiatric symptomology as longitudinally followed by PHQ-9 and GAD-7 scores. *Conclusion*. Optimal evaluation and management of mental illness in patients with complex neurologic symptomology may require independent evaluation and treatment by psychiatrists when clinically appropriate.

## 1. Background

Charles Bonnet syndrome (CBS) is principally characterized by complex visual hallucinations and ocular pathology causing vision loss [1]. Other characteristics include insight into the unreality of the perceptions, absence of mental disorders, and preserved cognitive status [2]. Cognitive impairment, stroke, or early Alzheimer's disease may be predisposing conditions. Furthermore, albeit the hallucinations being classically purely visual [3], a small minority of patients with CBS have reported concomitant auditory hallucinations. Patients with CBS, especially when having comorbid psychiatric symptomology and complex medical histories, may make diagnosis and treatment challenging. They may also often encounter significant mood symptoms more optimally addressed by psychiatrists.

## 2. Case Report

We present the case of a 66-year-old woman referred originally to Dartmouth-Hitchcock Epilepsy Center in 2010 for seizure disorder. In her 20s, she was involved in a motorcycle collision for which she was hospitalized for one week. No known traumatic brain injury (TBI) was diagnosed. Then she developed migraine headaches later, becoming more severe in her 30s. Also in her 30s and 40s, she developed fainting episodes. When she was standing for a long time, she would tumble down and get up. If she stood quickly, she would lose her vision. Those were diagnosed as syncope and were present all her life. It also seemed to run in her family.

Then, in 2007, she developed what she thought were seizures. They occurred when she was lying in bed. She would wake up and shake in the middle of the night. She would not lose consciousness. They were diagnosed as nonepileptic seizures (NES). Of note, the patient denied having any history of trauma, stressful life events, or other current stressors that might have triggered spells.

The patient initiated levetiracetam but nevertheless continued having events where she started whole-body shaking, lasting 5 to 10 minutes. She described having two recent events where she only had shaking of her right hand, arm, and face. After this event, her eyes were closed and she was very tired and slept for several hours. She had two of those right-sided events. All other events previously have included whole body shaking.

In the summer of 2008, she had an event where she was walking to the bathroom at night, she crashed down on the floor, had raccoon eyes and bruises, and was hospitalized elsewhere. There she had tilt table testing, which was positive and she was diagnosed with syncope; however, she was also noted to be hypertensive. She was not started on any medications during that outside hospitalization but was advised to sit at the edge of the bed and stand up slowly.

She also complained of having comprehension difficulties and poor memory, citing difficulties doing the laundry. At times, she was incapable of using a coffee maker that she has used for a long time and her husband confirmed that those events were more frequently occurring. She had not been sleeping very well and had difficulties concentrating. She denied feeling depressed. Physical exam found no cog-wheeling but falling diffusely with positive Romberg's and her eyes closed. She also had difficulties performing tandem gait but was able to walk on her heels and toes. The plan was for her to be admitted for video EEG (VEEG) monitoring and to have neuropsychological testing.

VEEG monitoring six weeks later in April 2010 captured multiple NES but no episodes with ictal correlates. She was discharged with outpatient follow-up outside our institution. A year later, in March of 2011, she re-presented with the chief complaints of headaches and did not have any significant NES spells. At this time, it was noted that while she did not have psychiatric care, she was taking both citalopram and clonazepam as prescribed by her primary care physician (PCP).

Half a year later in September 2011, the patient was seen with the chief complaint of headache as well as worsening depth perception. She was prescribed topiramate for her headaches and considered usage of amitriptyline, although patient was concerned about possibility of weight gain as had happened in the past. The patient was counseled on nonpharmacologic treatments of both anxiety and sleep hygiene. Nine months later in June 2012, the patient first described having more frequent falls as well as worsened headaches. For her symptoms, she was increased on topiramate prescribed hydroxyzine for moderate headaches and zolmatriptan for more severe headaches. The patient did not exhibit significant clinical improvement.

Then, in May 2013, the patient and her family described having hallucinations nearly every night, which consisted of the patient's witnessing other people talking to each other, but never to her directly. These people never talked to her or told her to do things. She denied these as the typical auditory hallucinations worrisome of primary thought disorders consisting of running commentary about the patient's own behavior. The auditory hallucinations were also never present in the absence of visual hallucinations. The visual hallucinations were nonhostile, but the patient's emotions associated with them were of fear due to uncertainty of the intentions of the people she was experiencing. However, she “knew that these people weren't really there” and that when she “turned on the lights, they would disappear.” She also described episodes where she was confused and had wandered about. This happened in late February and again early March 2013. Although she had been living in the same house for 37 years, it did not feel like the same house to her.

Visual symptoms included “seeing blue spots under my eyes on the left as well as on my hands.” Fundoscopic exam revealed floaters. The patient reported seeing cracks in the wall when looking at a white wall. The patient's husband reported that “she is becoming very forgetful.” These hallucinations were thought to be associated with nortriptyline, and dosage was reduced to 10 mg PO at night. It was first thought and documented at the end of May 2013 that the patient may have a neurodegenerative disease especially given significant atrophy of her frontal lobes as evidenced on magnetic resonance imaging (MRI). Four months later, her falls and headaches continued, although now she was “hearing voices that are not threatening.” The goal had been to decrease her nightly clonazepam dosage to see if this would allow hallucinations to improve. Of all her concerns, her headaches were causing the most misery.

At this point, referral to embedded psychiatric clinician within the neurology outpatient clinic occurred. The patient met our newly established quality improvement referral criteria by scoring above a certain threshold on depression screening. She was seen initially at the end of November, 2013. In addition to confirming her history above, it was found that family history was significant for early-onset Alzheimer's disease in the patient's brother. Physical exam did not find any cog-wheeling, rigidity, shuffling gait, or gait instability. Mental status exam revealed appearing younger than her stated age, anxious mood and flat affect, and thought content revealing the belief that “there are migrant French-Canadian workers at home threatening to hurt my family members.” She had seen them at least twice or thrice.

Her Montreal Cognitive Assessment (MOCA) score was 20/30 (score breakdown: 3/5 on visuospatial/executive: missed trails and did not get hands of clock correct; attention: 5/6: missed serial 7 subtraction; language: 2/3: did not repeat sentences perfectly with regard to pronouns, singular versus plural; delayed recall: 0/5, but recalled all five with category cuing; orientation: 5/6. Her PHQ-9 [4] and GAD-7 [5] scores were 7 and 9, resp.).

It was thought that it would be exceedingly rare, although not impossible, for the patient to have a new-onset psychosis at this age. However, the patient's description of her symptoms was not classically Charles Bonnet syndrome, as she experienced “real people talking and interacting” in addition to just seeing them. We decided to re-do MRI and obtain dementia workup, including B12/folate, and other basic labs, which were all unremarkable.

At our second appointment several months later, we again noticed the constellation of visual hallucinations, cognitive impairment, and history of multiple falls despite not having any clear Parkinsonian features on physical exam. At this appointment, both the patient and her family were more significantly distressed by especially the auditory hallucinations component of her experiences, described as “little people threatening to do things to me or my family.” Her PHQ-9 and GAD-7 scores had worsened to 10 and 17, respectively. MRI was unchanged compared to a year ago. It was thought at this time that the patient's distressing symptoms as well as psychotic symptoms could benefit from trial of quetiapine, to which the patient was naïve. Concurrently, we aimed to decrease clonazepam dosage from 3 mg to 2 mg per day over several weeks.

At our 3rd appointment, a month later, the patient reported that “everybody is noticing the difference with the Seroquel (quetiapine).” In fact, she described no longer having any AVH. Her quetiapine dosage had been titrated to effect to 200 mg PO QHS. Her PHQ-9 and GAD-7 scores were both zero. Of particular significance was also that the patient had tapered off clonazepam completely. As a result, both her headaches and her memory complaints had also decreased. The patient stated that since she was doing so well, she declined formal MOCA retesting.

However, at our 4th appointment, two months since the previous one, the patient described having horrible headaches and having hallucinations again, described as “I see people in the bathroom dressed in regular clothes, and they just stand there without talking to me.” The patient and her family did not seem to think that her headaches and hallucinations were connected. She also stated that sleep was awful despite taking hydroxyzine, melatonin, and quetiapine concurrently. We thus decided to transition her sertraline to venlafaxine using cross-titration.

At our 5th appointment a month and a half later, the patient had in the interim requested increasing quetiapine from 200 to 300 mg PO at night, and she was noticeably improved with both increase of quetiapine and transition from sertraline to venlafaxine. In fact, her headaches were “now gone completely” and her mood symptoms were also improved: “The new medication is a happy pill!” The patient described having minimal auditory and visual hallucinations which were “no longer bothering me.”

Approximately 8 months following her initial presentation, the patient described “things as going very well”: she was no longer waking up in the morning with any headaches and felt comfortable with seeing “little happy faces at night” when either falling asleep or waking up. At this point, the patient's venlafaxine dosage was 225 mg PO QDay and quetiapine dosage was between 300 and 400 mg PO at night. Given the patient's historical lack of depth perception and known history of floaters, it was thought that Charles Bonnet syndrome could be considered due to response of patient's AH to quetiapine but not her visual hallucinations. The patient was educated about the possibility that she had Charles Bonnet syndrome in addition to meeting criteria for major depressive disorder with Psychotic Features and she was reassured.

Ten to twelve months following her initial presentation, the patient described having a significant improvement in mood symptoms, and clinical exam and family collateral information indicated her Major Depression was in remission. The patient's medication dosages of venlafaxine 225 mg daily and 400 mg quetiapine at night were stable and did not cause any noticeable adverse effects, and discussion about eventually trying to find the minimally effective dose—especially in the case of the latter medication, due to concerns for potential metabolic and extrapyramidal adverse effects—ensued, although the patient and her family wanted to continue the current dosage given lack of any psychiatric symptoms.

## 3. Discussion

Our differential diagnosis was quite broad and included epilepsy, nonepileptic seizures (NES), depression, anxiety, pseudodementia, mild cognitive impairment/Alzheimer's dementia, Lewy-Body dementia (LBD), Parkinson's dementia, formal thought disorder/schizophrenia, headache, and Charles Bonnet syndrome.

The patient's history of documented NES most likely ruled out the development of new-onset epilepsy at the age of 70, although partial complex seizures could possibly account for her history of falls. However, the patient and her family's description of her NES made partial complex seizures less likely.

In line with the conceptualization of NES, there is a possibility that the patient's episodes of confusion and wandering alongside the feeling of unfamiliarity of her house might represent dissociative phenomena, specifically depersonalization, which may be associated with psychosocial stressors, although the patient was not able to clearly identify any known stressors.

When the patient had MOCA scores completed, pseudodementia was initially considered given severity of documented depression and anxiety. However, the patient's ability to perform the majority of her ADLs and IADLs as well as having no clinically apparent anomia, aphasia, or apraxia made mild cognitive impairment or Alzheimer's less likely.

Once the patient was found to have memory and cognitive impairment alongside fall frequency, the diagnosis of Lewy-Body Dementia was then considered. However, of the clinical symptomatic triad of fluctuating cognitive impairment, extrapyramidal features, and visual hallucinations, the patient primarily had only the latter once her clonazepam was tapered and discontinued, making LBD less likely. Furthermore, the patient did not demonstrate any extrapyramidal symptoms or clinical sensitivity to quetiapine, an atypical antipsychotic, which definitively goes against the diagnosis of LBD.

The patient's history of frequent falls and residual daytime grogginess was most likely attributable to her usage of nighttime clonazepam. Her history of myriad falls was initially thought to be due to a combination of either NES or syncope, both previously diagnosed, and thus their attribution to Parkinsonism was thought less likely. Much less likely was that the patient presented with 1st onset formal thought disorder at the age of 70.

The patient's once or twice monthly to near daily chronic headaches or transformed migraines were associated with “flashing lights,” and so the notion that her vision was compromised at baseline (“problems with depth perception”) was not on the forefront of our conceptualization towards the possibility of Charles Bonnet Syndrome until much later in her clinical course.

The patient's comorbid mood and anxiety symptoms as well as her preexisting diagnosis of NES made her psychiatric diagnostic formulation particularly challenging. Furthermore, while paranoid delusions are very rarely encountered in patients with CBS, they have been known to be associated with the visual hallucinations experienced [6]. In our patient who was eventually tentatively diagnosed with several comorbid neurological diagnoses—including headache, nonepileptic seizures, and Charles Bonnet syndrome—attributing her psychiatric symptomology parsimoniously to her known neurological diagnoses provided context for psychopharmacologic treatment.

It was thought that the patient's cognitive status, auditory hallucinations, and paranoid ideation were unlikely attributable to simply a diagnosis of Charles Bonnet syndrome. Rather, these clinical features indicated the severity of her psychiatric symptoms such that the patient met diagnostic criteria for major depressive disorder with Psychotic Features.

Our case therefore illustrates the evolution of a patient's symptomology and ultimate benefit of formal psychiatric management. Specifically, venlafaxine ameliorated our patient's anxiety, depression, and headache—whether FDA-approved (anxiety and depression) or off-label (in the case of headache). Quetiapine targeted the amelioration of her anxiety and AVH. Equally noteworthy was that the patient exhibited dramatic improvement following taper and eventual discontinuation of clonazepam.

Given that the medication received by the patient was complex and evolved, please refer to Table 1—a chronological synopsis of signs and symptoms, tentative diagnoses, medication changes, and responses to psychopharmacologic treatment—in order to clarify any drug-related side-effects.

## 4. Conclusion

Referral of this patient with complex neurological history to a co-located psychiatrist within the neurology department guided the diagnostic formulation and eventual diagnoses of the patient's underlying medical and psychiatric comorbidities. Therefore, optimal evaluation and management of mental illness in patients with complex neurological symptoms may require independent treatment by psychiatrists when clinically appropriate. Furthermore, psychiatric comorbidities of patients with neurological disorders may be more optimally addressed by dedicated psychiatrists co-located within neurology clinics in order to reduce utilization and prevent avoidable reencounters [7, 8]. We advocate for more standardized referral procedures including baseline and longitudinal screening of psychiatric comorbidity using validated instruments for patients seen in neurology clinics.

## Figures and Tables

**Table 1:** Chronological synopsis of signs and symptoms, tentative diagnoses, medication changes, and responses to psychopharmacologic treatment.

Date	Signs and symptoms	[Tentative diagnoses] and (differential diagnoses)	Psychopharmacologic and nonpsychopharmacologic treatments, if any	Responses to treatments, if any
1970s	Motorcycle collision requiring one week's hospitalization. No known TBI was diagnosed. Note the large time lag between the collision and the symptoms that follow.	[Postconcussive headache]		

1980s	Severe unilateral headaches	[Migraines]	Amitriptyline	Partial effective response, but patient discontinued it due to significant weight gain

1990s	Fainting episodes. When the patient stood for a long time, she would tumble down and get up. If she stood quickly, she would lose her vision.	[Syncope—appeared to be familial]		

2007	Thought to be having seizures. These occurred when the patient was lying in bed. She would wake up and shake in the middle of the night. No loss of consciousness.	[Epilepsy versus nonepileptic seizures]	Levetiracetam	Continued having events where the patient had whole-body shaking, lasting 5 to 10 minutes and also shaking of her right hand, arm, and face. Following events, eyes were closed and she was very tired and slept for several hours. She had at least two of the right-sided events.

2008 Summer	Had an event where she was walking to the bathroom at night and crashed down on the floor; she had raccoon eyes and bruises and was hospitalized	[Syncope diagnosed by tilt-table testing; simultaneously noted to be hypertensive]	Not started on any medications during that hospitalization but advised to sit at the edge of the bed and stand up slowly.	

2008 Fall	First complained of having comprehension difficulties and poor memory, citing difficulties doing the laundry. At times, she was incapable of using a coffee maker that she has used for a long time and her husband confirmed that those events were more frequently occurring. She had not been sleeping very well and had difficulties concentrating. She denied feeling depressed. Physical exam found no cog-wheeling but falling diffusely with positive Romberg's and her eyes closed. She also had difficulties performing tandem gait but was able to walk on her heels and toes.	[Pseudodementia versus dissociative state versus adverse effect to treatment with clonazepam]	Was on clonazepam during this time	

April 2010	VEEG monitoring captured only NES.	[NES]	None	

March 2011	Patient re-presented in outpatient clinic with chief complaint of headaches and did not have any significant NES spells.	[Headaches; NES]	While she did not have psychiatric care, she initiated both citalopram and clonazepam as prescribed by her primary care physician (PCP).	Continued to have falling episodes, dissociative episodes.

September 2011	Seen in outpatient neurology clinic with chief complaint of headache and worsening depth perception	[Headaches; NES]	Prescribed topiramate for headaches and considered amitriptyline, although patient was concerned about possibility of weight gain as had happened in the past. The patient was counseled on nonpharmacologic treatments of both anxiety and sleep hygiene.	

June 2012	First described having more frequent falls as well as worsened headaches	[Headaches; NES]	Increased on topiramate and initiated hydroxyzine for moderate headaches and zolmatriptan for more severe headaches.	Did not exhibit significant clinical improvement

March to May 2013	Nightly hallucinations consisting of witnessing other people talking to each other, but never to her directly. These people never talked to her or told her to do things. She denied these as the typical AHs worrisome of primary thought disorders consisting of running commentary about the patient's own behavior. The AHs were also never present in the absence of VHs. The VHs were nonhostile, but the patient's emotions associated with them were of fear due to uncertainty of the intentions of the people she was experiencing. However, she “knew that these people weren't really there” and that when she “turned on the lights, they would disappear.” She also described episodes where she was confused and had wandered about. Although she had been living in the same house for 37 years, it did not feel like the same house to her.	[Dissociative episodes, Major Depression with Psychotic Features] (schizophrenia)	The patient and her family thought that the hallucinations were associated with nortriptyline, and dosage was reduced to 10 mg PO at night	No significant changes from reduction in nortriptyline

November 2013	Scored PHQ-9 greater than 15 to be considered for evaluation with embedded psychiatric clinician within neurology department. Physical exam did not find any cog-wheeling, rigidity, shuffling gait, or gait instability. MSE revealed appearing younger than her stated age, anxious mood and flat affect, and thought content revealing the belief that “there are migrant French-Canadian workers at home threatening to hurt my family members.” She had seen them at least twice or thrice. MOCA score of 20/30.	[Pseudodementia], (Alzheimer's, Charles Bonnet syndrome)	Re-did MRI and obtained dementia workup, including B12/folate, and other basic labs, which were all unremarkable.	

January 2014	Had constellation of visual hallucinations, cognitive impairment, and history of multiple falls despite not having any clear Parkinsonian features on physical exam. Both the patient and her family were more significantly distressed by especially the AH component of her experiences, described as “little people threatening to do things to me or my family.” Worsening PHQ-9 and GAD-7 scores. MRI was unchanged compared to a year ago.	[Major Depression with Psychotic Features, adverse effect of clonazepam], (Lewy-Body dementia, Alzheimer's, and Primary Thought Disorder)	It was thought at this time that the patient's distressing symptoms as well as psychotic symptoms could benefit from trial of quetiapine, to which the patient was naïve. Concurrently, we aimed to decrease clonazepam dosage from 3 mg to 2 mg per day over several weeks.	

February 2014	“Everybody is noticing the difference with the Seroquel (quetiapine).” No longer had any AVH. Her quetiapine dosage had been titrated to effect to 200 mg PO QHS. Her PHQ-9 and GAD-7 scores were both 0. As a result, the patient stated that since she was doing so well, she declined formal MOCA retesting.	[Major Depression with Psychotic Features, in partial remission; medication-induced falls and daytime grogginess and impaired concentration associated with clonazepam]	Quetiapine titrated to effect to 200 mg PO QHS. Patient had also tapered off clonazepam completely.	Both headaches and her memory complaints had also decreased.

April 2014	Described having horrible headaches and having hallucinations again: “I see people in the bathroom dressed in regular clothes, and they just stand there without talking to me.” The patient and her family did not seem to think that her headaches and hallucinations were connected. She also stated that sleep was awful despite taking hydroxyzine, melatonin, and quetiapine concurrently.	[Major Depression with Psychotic Features; Charles Bonnet syndrome]	Transitioned sertraline to venlafaxine using cross-titration.	

May 2014	Headaches were “now gone completely” and her mood symptoms were also improved: “The new medication is a happy pill!” The patient described having minimal auditory and visual hallucinations which were “no longer bothering me.”	[Major Depression with Psychotic Features, in remission; migraines, currently in remission; Charles Bonnet syndrome]	Patient and family had requested trial of increasing quetiapine from 200 to 300 mg PO at night; titrated venlafaxine up to 225 mg PO daily.	Noticeable improvement with both increases of quetiapine and transition from sertraline to venlafaxine

June 2014	“Things are going very well”: she was no longer waking up in the morning with any headaches and felt comfortable with seeing “little happy faces at night” when either falling asleep or waking up. Given the patient's historical lack of depth perception and known history of floaters, it was thought that Charles Bonnet syndrome could be considered due to response of patient's AH to quetiapine but not her visual hallucinations.	[Charles Bonnet syndrome; Major Depression with Psychotic Features]	At this point, the patient's venlafaxine dosage was 225 mg PO QDay and quetiapine dosage was between 300 and 400 mg PO at night. The patient was educated about the possibility that she had Charles Bonnet syndrome in addition to meeting criteria for major depressive disorder with Psychotic Features and she was reassured.	

September 2014	Significant improvement in mood symptoms, and clinical exam and family collateral information indicated her Major Depression was in remission. The patient's medication dosages of venlafaxine 225 mg daily and 400 mg quetiapine at night were stable and did not cause any noticeable adverse effects. The patient was no longer distressed by the VH.	[Charles Bonnet syndrome; Major Depression with Psychotic Features in remission]	Discussion eventually trying to find the minimally effective dose—especially in the case of quetiapine, due to concerns for potential metabolic and extrapyramidal adverse effects. However, the patient and her family desired continuation of her current medications and dosages given lack of any psychiatric symptoms.	
